# XBP1-Independent UPR Pathways Suppress C/EBP-β Mediated Chondrocyte Differentiation in ER-Stress Related Skeletal Disease

**DOI:** 10.1371/journal.pgen.1005505

**Published:** 2015-09-15

**Authors:** Trevor L. Cameron, Katrina M. Bell, Irma L. Gresshoff, Lisa Sampurno, Lorna Mullan, Joerg Ermann, Laurie H. Glimcher, Raymond P. Boot-Handford, John F. Bateman

**Affiliations:** 1 Murdoch Childrens Research Institute, Parkville, Victoria, Australia; 2 Wellcome Trust Centre for Cell-Matrix Research, Faculty of Life Sciences, University of Manchester, Manchester, United Kingdom; 3 Department of Immunology and Infectious Diseases, Harvard School of Public Health, Boston, Massachusetts, United States of America; 4 Weill Cornell Medical College, Cornell University, New York, New York, United States of America; 5 Department of Biochemistry and Molecular Biology, University of Melbourne, Parkville, Victoria, Australia; Stanford University School of Medicine, United States of America

## Abstract

Schmid metaphyseal chondrodysplasia (MCDS) involves dwarfism and growth plate cartilage hypertrophic zone expansion resulting from dominant mutations in the hypertrophic zone collagen, *Col10a1*. Mouse models phenocopying MCDS through the expression of an exogenous misfolding protein in the endoplasmic reticulum (ER) in hypertrophic chondrocytes have demonstrated the central importance of ER stress in the pathology of MCDS. The resultant unfolded protein response (UPR) in affected chondrocytes involved activation of canonical ER stress sensors, IRE1, ATF6, and PERK with the downstream effect of disrupted chondrocyte differentiation. Here, we investigated the role of the highly conserved IRE1/XBP1 pathway in the pathology of MCDS. Mice with a MCDS collagen X p.N617K knock-in mutation (*ColX* ^*N617K*^) were crossed with mice in which *Xbp1* was inactivated specifically in cartilage (*Xbp1* ^*CartΔEx2*^), generating the compound mutant, *C/X*. The severity of dwarfism and hypertrophic zone expansion in *C/X* did not differ significantly from *ColX* ^*N617K*^, revealing surprising redundancy for the IRE1/XBP1 UPR pathway in the pathology of MCDS. Transcriptomic analyses of hypertrophic zone cartilage identified differentially expressed gene cohorts in MCDS that are pathologically relevant (XBP1-independent) or pathologically redundant (XBP1-dependent). XBP1-independent gene expression changes included large-scale transcriptional attenuation of genes encoding secreted proteins and disrupted differentiation from proliferative to hypertrophic chondrocytes. Moreover, these changes were consistent with disruption of C/EBP-β, a master regulator of chondrocyte differentiation, by CHOP, a transcription factor downstream of PERK that inhibits C/EBP proteins, and down-regulation of C/EBP-β transcriptional co-factors, GADD45-β and RUNX2. Thus we propose that the pathology of MCDS is underpinned by XBP1 independent UPR-induced dysregulation of C/EBP-β-mediated chondrocyte differentiation. Our data suggest that modulation of C/EBP-β activity in MCDS chondrocytes may offer therapeutic opportunities.

## Author Summary

A significant component of the molecular pathology of many inherited skeletal disorders caused by mutations that cause misfolding and intracellular retention of extracellular matrix proteins is the induction of a cellular response to endoplasmic reticulum stress called the unfolded protein response (UPR). In the case of Schmid metaphyseal chondrodysplasia (MCDS) caused by collagen X misfolding mutations, the consequences of the UPR have been shown to be the central cause of the cartilage pathology. Thus understanding the involvement of canonical UPR sensors, IRE1, ATF6, and PERK and their downstream signalling effects on chondrocyte differentiation and function is important for defining disease mechanisms and devising new therapies. Using a mouse model expressing misfolding collagen X and lacking IRE1/XBP1 pathway activity in chondrocytes, we demonstrate that this highly conserved UPR pathway is redundant to the cartilage pathology thus implicating XBP1-independent UPR signalling pathways. Based on detailed analysis of gene expression patterns we propose that XBP1-independent UPR driven disruption of C/EBP-β, a master regulator of chondrocyte differentiation, is important for the pathophysiology. Strategies designed to modulate C/EBP-β activity may thus offer therapeutic opportunities.

## Introduction

Longitudinal growth of endochondral bones occurs bi-directionally under the control of cartilaginous growth plates located at each end of growing bones. In the growth plate, the extracellular matrix of cartilage is synthesized and remodelled by chondrocytes undergoing differentiation involving proliferation and hypertrophy, before being remodelled, calcified, and vascularised to produce primary bone [1]. As with other professional secretory cells, chondrocytes rely on the maintenance of endoplasmic reticulum (ER) homeostasis through molecular pathways that regulate protein folding quality control [2]. Known collectively as the unfolded protein response (UPR), these pathways alleviate ER stress by enhancing the protein folding capacity of the ER, by up-regulating protein degradation machinery such as the ER-associated degradation (ERAD) pathway, or by regulating the translation or half-life of transcripts encoding secreted proteins. The UPR is activated by ER membrane-spanning sensors, including activating transcription factor 6 (ATF6), PRKR-like endoplasmic reticulum kinase (PERK), and inositol-requiring enzyme-1 (IRE1), which detect the accumulation of misfolded proteins in the ER via their lumenal domains, and transmit this information to activate downstream signalling pathways via their cytoplasmic domains [3–5].

Activated ATF6 translocates to the Golgi apparatus, where it is proteolytically cleaved by site 1 protease to produce a 50kDa transcription factor that targets genes harbouring ERSE elements in their promoters, such as the molecular chaperone BiP [6]. PERK is a kinase that catalyses the phosphorylation of eIF2α in response to ER stress, down-regulating overall translation by inhibiting assembly of the translational initiation complex [7], and driving the increased translation of specific transcripts including activating transcription factor 4 (ATF4) [8,9]. Activated IRE1 splices a 26nt fragment from the coding sequence of the X-box binding protein 1 (*XBP1*) mRNA to encode a transcription factor responsible for the expression of multiple UPR target genes [10].

While the UPR exists primarily as an adaptive mechanism to accommodate relatively minor fluctuations in protein misfolding during normal cellular function, it may also have deleterious consequences when chronically activated, as occurs in pathologies characterized by constitutive expression of mutant, misfolding proteins. We demonstrated in two mouse models of Schmid metaphyseal chondrodysplasia (MCDS), a condition involving dwarfism and growth plate hypertrophic zone expansion caused by autosomal dominant mutations in the hypertrophic zone marker collagen X (*Col10a1*), that hypertrophic zone ER stress *per se* is sufficient to phenocopy the disease [11]. Using transcriptional profiling approaches, we characterised the UPR deployed by chondrocytes under such stress *in vivo* [12]. Our work revealed that ATF6, PERK, and IRE1 are each activated, that components of the ERAD pathway are up-regulated, and that although this response enables the chondrocytes to survive the stress, it also results in disrupted differentiation and developmental arrest in a proliferative chondrocyte-like state [12].

Since IRE1/XBP1 regulates the most highly conserved of the UPR pathways, present in all eukaryotes from yeast through to higher vertebrates [4], and is a key factor in the pathology of numerous diseases involving ER stress [13], we sought to address its influence on the pathology of MCDS. Thus, we crossed our collagen X p.Asn617Lys knock-in mouse model of MCDS (*ColX* ^*N617K*^) [11] with our *Xbp1* ^*CartΔEx2*^ mouse, in which XBP1 activity is ablated specifically from chondrocytes [14], to generate the compound mutant, *ColX* ^*N617K*^ */Xbp1* ^*CartΔEx2*^ (*C/X*). Here we demonstrate surprising redundancy for the IRE1/XBP1 pathway in the MCDS UPR by showing that the pathology of the *ColX* ^*N617K*^ mouse is not substantially altered by the inactivation of XBP1 in *C/X*. Through transcriptional profiling of mutant and wildtype hypertrophic zones, we separate genes whose expression is regulated in the chondrocyte UPR in an XBP1-dependent manner from those regulated independently of XBP1. Since XBP1 is redundant in the pathology of MCDS, we focus on genes regulated independently of XBP1 in order to identify those that are central to the pathology of the disease. We demonstrate that for chondrocytes, the XBP1-independent components of the UPR up-regulate gene networks associated with the ER and translation of mRNA, and down-regulate genes encoding glycoproteins and components of the extracellular matrix, as well as those associated with angiogenesis and skeletal system development. Thus we suggest that the hypertrophic zone expansion and delayed ossification observed in MCDS results from XBP1-independent transcriptional suppression of genes involved in cartilage matrix turnover, vascular invasion, and growth plate ossification. We propose interaction between C/EBP homologous protein (CHOP), a transcription factor up-regulated downstream of PERK in response to ER stress with known roles in regulating apoptosis and cell differentiation [15] and CCAAT/enhancer binding protein beta (C/EBP-β), a transcription factor important for the transition from chondrocyte proliferation to hypertrophy [16–18], as a key point at which the UPR and chondrocyte differentiation machinery intersect in MCDS.

This is the first study to present evidence supporting a direct link between the UPR and a blockage in cell differentiation mediated by transcriptional suppression of C/EBP-β in a mouse model of human disease. Moreover, it establishes a rational foundation for future studies investigating both C/EBP-β as a potential therapeutic target in the treatment of MCDS, and the possible role of disruption to differentiation pathways controlled by C/EBP-β in other ER stress-associated human disease contexts.

## Results

### Generation of MCDS mice where XBP1 is functionally inactivated in cartilage

**Fig 1:**
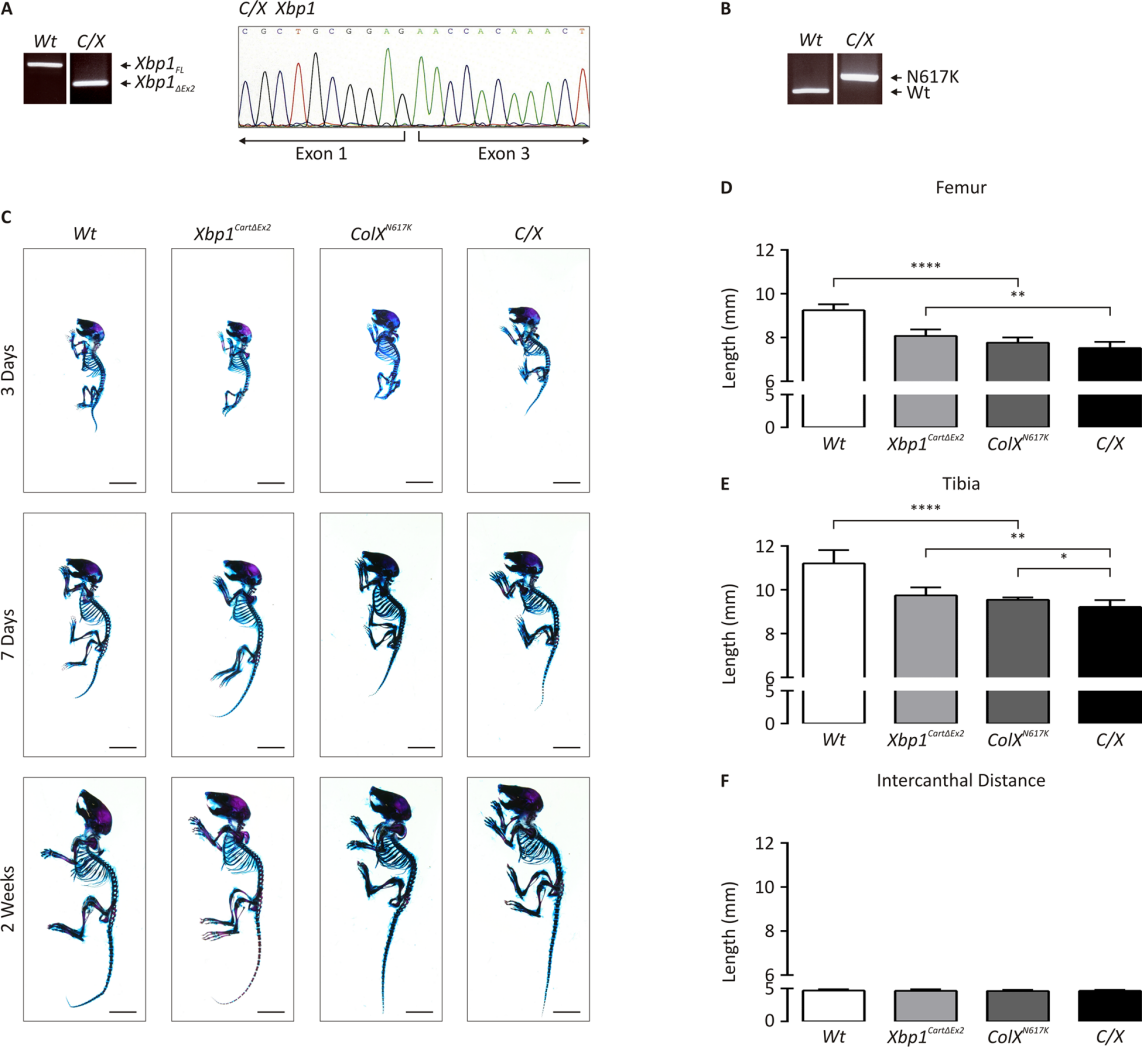
Genetic and morphometric characterization of *C/X* mice. *(A)* RT-PCR on cDNA derived from femoral epiphyseal cartilage from wildtype (*Wt*) and *C/X* to detect the full-length form of *Xbp1* (*Xbp1*_*FL*_) or the inactive form of *Xbp1*, lacking exon 2 (*Xbp1*_*ΔEx2*_), and sequencing of cDNA from *C/X* femoral head cartilage to assay for the deletion of *Xbp1* exon 2. *(B)* PCR for residual *loxP* site downstream of the p.Asn617Lys *Col10a1* coding sequence using genomic DNA derived from *Wt* and *C/X*. *(C)* Alizarin red S/Alcian blue staining of skeletal preparations from newborn, 1 week, and 2 week wildtype (*Wt*), *Xbp1* ^*CartΔEx2*^, *ColX* ^*N617K*^ and *C/X* mice. *(D-F)* Quantification of *(D)* femoral and *(E)* tibial length, and *(F)* intercanthal distance (ICD) from legs from 2 week *Wt* and mutant mice–*Wt*, N = 8; *Xbp1* ^*CartΔEx2*^, N = 8, *ColX* ^*N617K*^, N = 6; *C/X*, N = 8; statistical analysis performed using Student’s *t* test.

We crossed our collagen X p.Asn617Lys knock-in mouse model of MCDS (*ColX* ^*N617K*^) [11] with mice in which *Col2a1*-Cre/*loxP*-mediated deletion of *Xbp1* exon 2 renders *Xbp1* completely inactive specifically in chondrocytes (*Xbp1* ^*CartΔEx2*^) [14], to generate *ColX* ^*N617K*^ */Xbp1* ^*CartΔEx2*^ (*C/X*). *C/X* mice were viable, fertile, and bred normally. RT-PCR and sequencing analysis of cDNA derived from femoral head cartilage of 14 day old *C/X* and wildtype mice confirmed the complete inactivation of XBP1 by Cre/*loxP*-mediated deletion of *Xbp1* exon 2 in the mutant (Fig 1A). PCR on genomic DNA derived from *C/X* and wildtype tail lysates revealed the homozygous presence of the collagen X p.Asn617Lys allele in the mutant, identifiable due to the presence of a residual *loxP* site downstream of the *Col10a1* coding sequence remaining from the gene targeting construct used to create the *ColX* ^*N617K*^ mouse from which *C/X* was derived (Fig 1B).

### Neither dwarfism nor the hypertrophic zone expansion of *ColX* ^*N617K*^ is significantly altered by loss of XBP1 activity in *C/X* chondrocytes

To determine the impact of XBP1-dependent UPR signaling in the pathology of MCDS, we used morphometric and histological approaches to compare the skeletal phenotypes of wildtype, *ColX* ^*N617K*^, *Xbp1* ^*CartΔEx2*^, and *C/X* mice. Skeletal preparations of newborn, seven day old, and two week old mutant and wildtype mice were stained with Alcian blue and Alizarin red to visualize cartilage and bone. Although no overt phenotype was apparent by visual inspection (Fig 1C) quantitative analysis of individual skeletal elements from two week old animals indicated significant reductions in the length of endochondral bones (tibiae and femora) when *ColX* ^*N617K*^ was compared to wildtype, as previously reported [11], and also when *C/X* was compared with *Xbp1* ^*CartΔEx2*^ (Fig 1D and 1E). When skeletal elements from *C/X* were compared with *ColX* ^*N617K*^ however, there was no significant difference in femoral length, while the tibial length was found to be only very modestly reduced in *C/X* compared with *Col* ^*N617K*^. No difference was observed in intramembranous bone growth (as approximated by intercanthal distance measurements) between any of the mutants compared with wildtype (Fig 1F).

**Fig 2:**
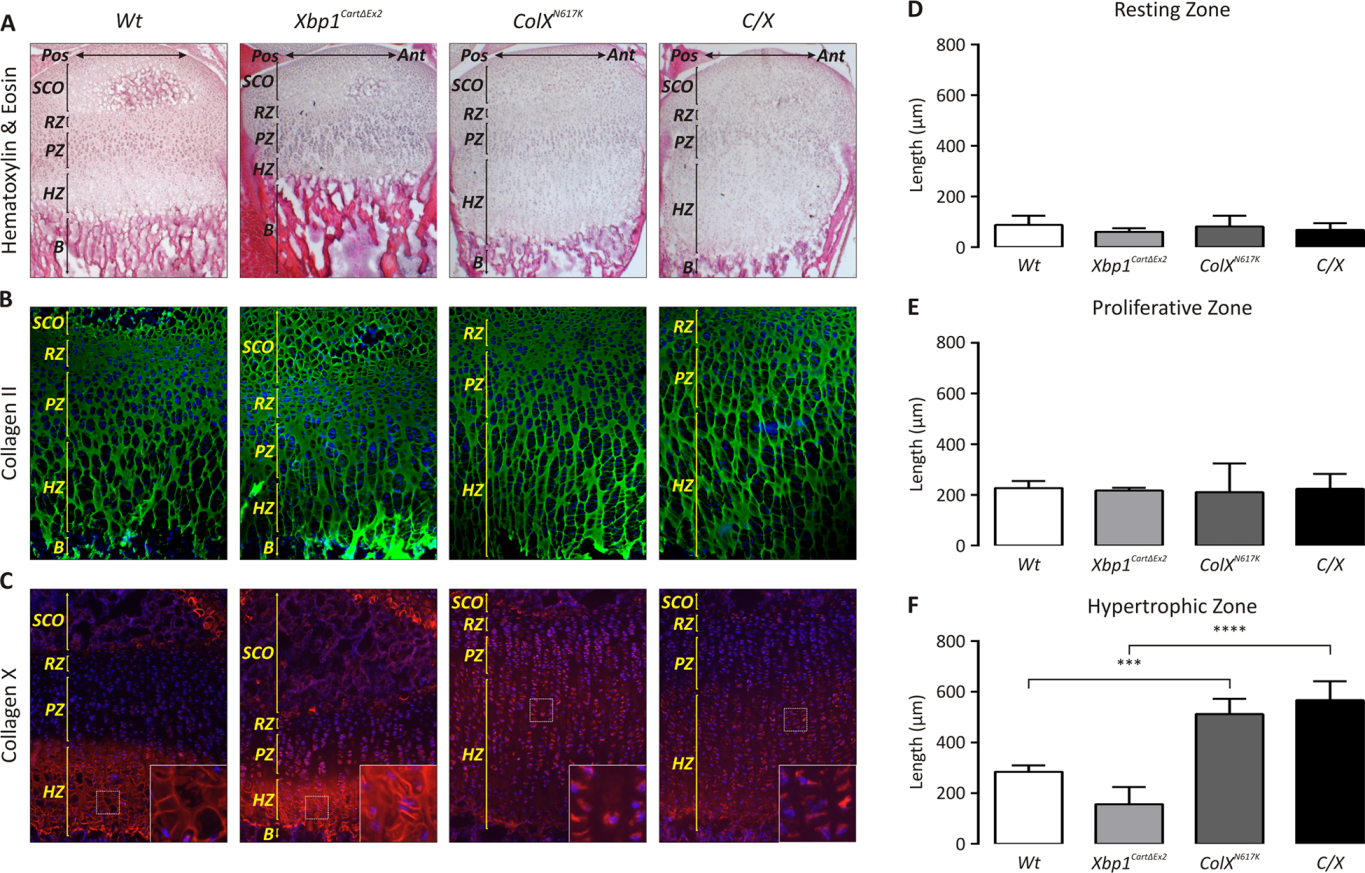
Ablation of XBP1 does not significantly affect the MCDS phenotype in *C/X* mice. *(A-C)* Tibial epiphyseal cryosections from 2 week *Wt*, *Xbp1* ^*CartΔEx2*^, *ColX* ^*N617K*^ and *C/X* mice stained with *(A)* haematoxylin and eosin (H&E), or by immunofluorescence using *(B)* anti-collagen II or *(C)* anti-collagen X antibodies; B—Bone; HZ—Hypertrophic Zone; PZ—Proliferative Zone; SCO—Secondary Center of Ossification. *(D-F)* Quantification of growth plate *(D)* resting zone, *(E)* proliferative zone, and *(F)* hypertrophic zone lengths in mutant and *Wt* mice; N = 3 for each genotype; statistical analysis performed using Student’s *t* test.

**Fig 3:**
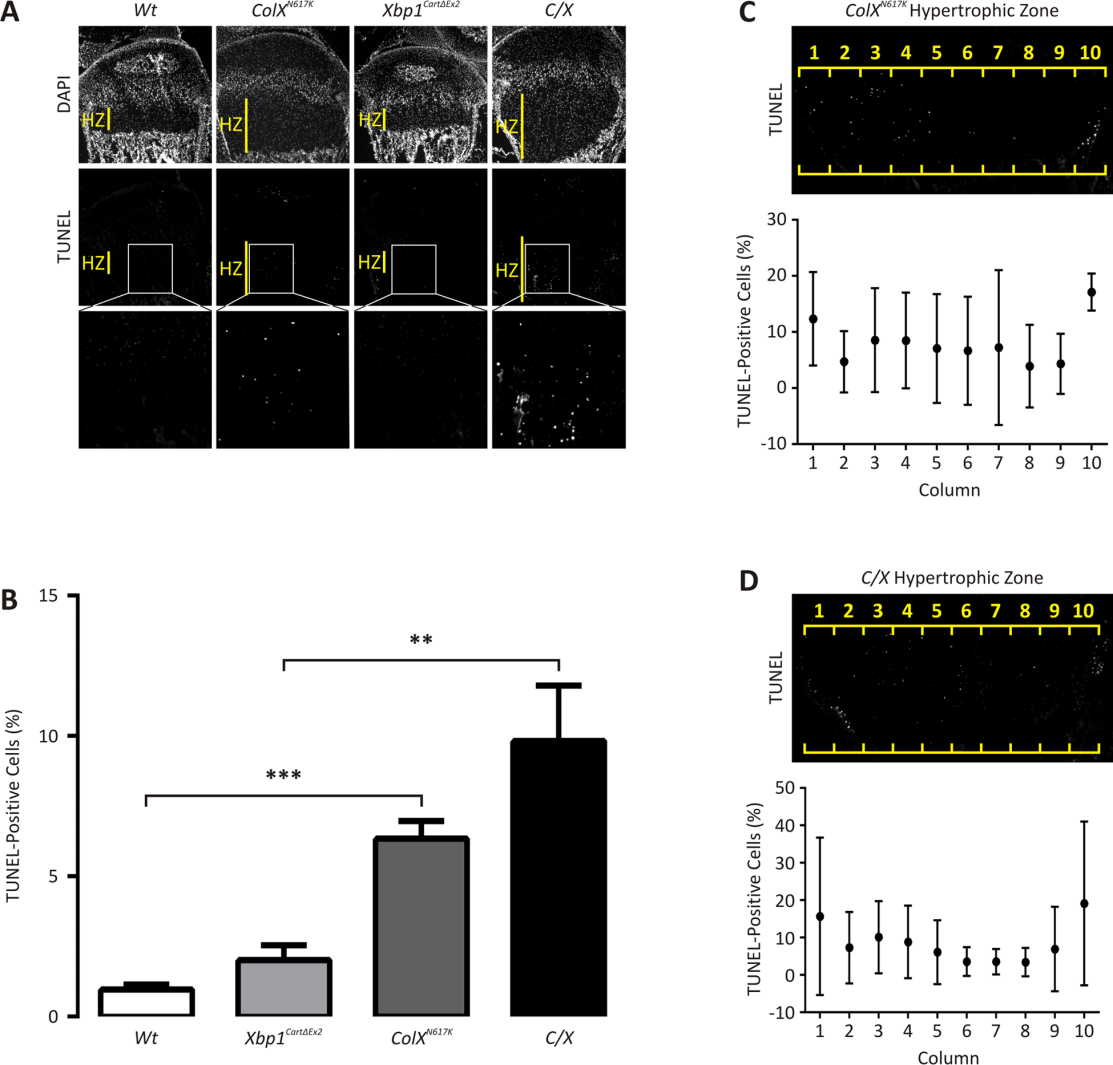
Apoptosis is elevated in 2 week *ColX* ^*N617K*^ and *C/X* growth plate cartilage. *(A)* Representative 2 week wildtype (*Wt*), *Xbp1* ^*CartΔEx2*^, *ColX* ^*N617K*^ and *C/X* tibial growth plate sagittal cryosections analysed by TUNEL with DAPI counterstaining; HZ—hypertrophic zone. Boxes inset indicate magnified areas of the hypertrophic zones containing TUNEL-positive chondrocytes. *(B)* TUNEL analysis of at least 6 tibial growth plate sections from each of 3 *Wt*, *Xbp1* ^*CartΔEx2*^, *ColX* ^*N617K*^, and *C/X* mice, expressed as the number of TUNEL-positive chondrocytes in the hypertrophic zone as a percentage of the total number of chondrocytes per zone (as defined by DAPI-stained nuclei), and showing standard deviation around the mean. *(C*,*D)* Representative 2 week *(C) ColX* ^*N617K*^ and *(D) C/X* tibial growth plate cryosections, showing the distribution of TUNEL-positive cells along the antero-posterior axis of *ColX* ^*N617K*^ and *C/X* hypertrophic zones, as demarcated by 10 consecutive columns (1–10) of arbitrary width. Plots depict the number of TUNEL-positive chondrocytes in each column as a percentage of the total number of chondrocytes per column (as defined by DAPI-stained nuclei), from the same *ColX* ^*N617K*^ and *C/X* mice as analysed in *(B)*, and showing standard deviation around the mean. Statistical analysis performed using Student’s t-test, ** *p* < 0.01, *** *p* < 0.001.

Growth plate sections from each strain were analyzed histologically by H&E staining (Fig 2A), and by immunofluorescence with antibodies for cartilage-specific collagen II (Fig 2B) to visualize the organization and extent of the growth plate cartilage extracellular matrix, and collagen X to demarcate the hypertrophic zone of the growth plate (Fig 2C). Using H&E-stained sections to perform quantitative analyses of growth plate zone lengths between our various mouse strains, we found there was no significant difference between the length of the pathologically expanded hypertrophic zones observed in *ColX* ^*N617K*^ [11] and *C/X* (Fig 2D–2F). Consistently however, we observed a progressive increase in the severity of hypertrophic zone expansion in the *C/X* mice from the anterior to posterior margin of the growth plate, whereas the severity of hypertrophic zone expansion was unchanged across this gradient in *ColX* ^*N617K*^ (Figs 2A and 3A). No obvious difference in the abundance and organization of collagen II in the extracellular matrix was apparent between each mutant and wildtype. Collagen X staining was reduced and largely intracellular in both *ColX* ^*N617K*^ and *C/X* hypertrophic zones reflecting previously described reduced secretion of the mutant misfolded collagen X and its increased intracellular degradation by the ER-associated proteasomal degradation pathway [11,12].

These morphometric and histological data indicate that the severity of the dwarfism caused by expression of the p.N617K collagen X in *ColX* ^*N617K*^ mice was not substantially altered by loss of XBP1 activity in *C/X*, revealing surprising redundancy for the IRE1/XBP1 pathway in the pathology of MCDS, and implying that XBP1-independent consequences of collagen X-induced ER stress must underpin the disease pathology.

### ER stress-induced apoptosis is regulated independently of XBP1

TUNEL analysis was conducted on 14 day old wildtype, *ColX* ^*N617K*^, *Xbp1* ^*CartΔEx2*^, and *C/X* tibial growth plates to determine whether loss of XBP1 from chondrocytes would alter cell fate during ER stress (Fig 3A). The rate of apoptosis in each mouse was quantified by determining the extent of apoptosis as a percentage of the total number of chondrocytes in the zones (Fig 3B). As expected the percentage of apoptotic cells observed in the hypertrophic zones of wildtype and *Xbp1* ^*CartΔEx2*^ growth plates was low, typically in the order of 1–2% as previously described [14]. Significantly more apoptotic cells were detected in *ColX* ^*N617K*^ compared with wildtype, and in *C/X* compared with *Xbp1* ^*CartΔEx2*^. While a trend towards increased apoptosis was observed in the hypertrophic zones of *C/X* versus *ColX* ^*N617K*^, the difference was not statistically significant. Thus our data suggest that apoptosis is a feature of the pathology of MCDS, occurring in chondrocytes in the *ColX* ^*N617K*^ and *C/X* hypertrophic zones by two weeks of age, independently of XBP1 signaling.

To explore whether the apparent antero-posterior gradient of hypertrophic zone expansion in *C/X* is related to cell death, we set out to quantify the lateral distribution of apoptotic cells in the hypertrophic zones of the collagen X mutant growth plates. Thus, we divided the hypertrophic zones of *ColX* ^*N617K*^ and *C/X* growth plates into 10 columns of arbitrary width and scored the number of TUNEL-positive chondrocytes in each column, again normalised against the total number of cells per column (Fig 3C and 3D). The distribution of apoptotic cells was found to be asymmetrical across the width of the *ColX* ^*N617K*^ (Fig 3C) and *C/X* (Fig 3D) hypertrophic zones with the highest percentages of apoptotic chondrocytes generally found in the peripheral-most columns in both mutants, at both the anterior and posterior margins of the growth plate. Thus, the antero-posterior gradient of hypertrophic zone expansion observed in *C/X* growth plates does not correlate with the extent of apoptosis observed in these tissues.

### Expression profiling of mutant and wildtype hypertrophic zone RNA distinguishes XBP1-independent gene cohorts from XBP1-dependent gene cohorts

**Fig 4:**
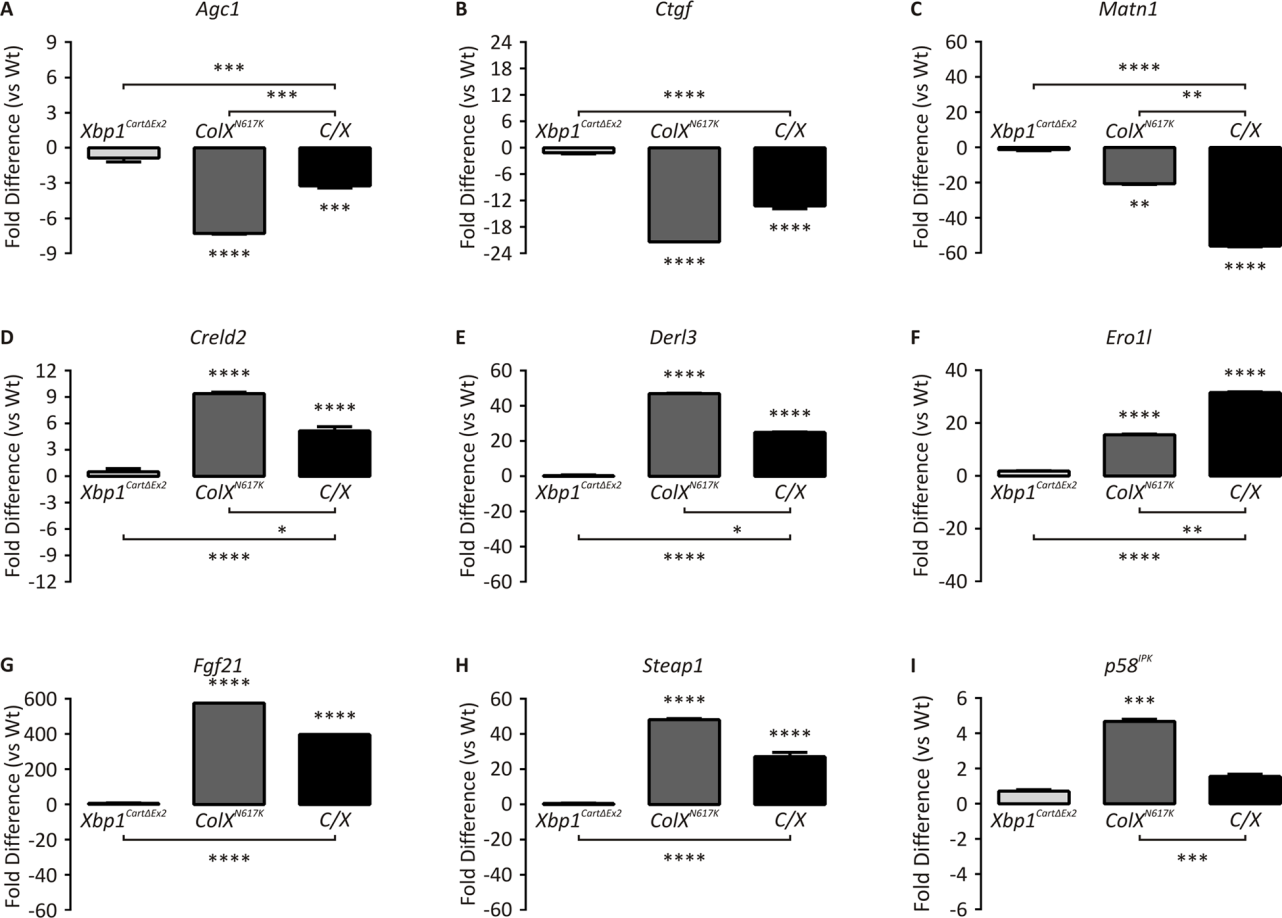
Quantitative PCR of mutant and wildtype hypertrophic zones. qPCR with primers specific for *(A) Agc1*, *(B) Ctgf*, *(C) Matn1*, *(D) Creld2*, *(E) Derl3*, *(F) Ero1l*, *(G) Fgf21*, *(H) Steap1*, and *(I) p58IPK* on cDNA derived from *Wt*, *Xbp1* ^*CartΔEx2*^, *ColX* ^*N617K*^ and *C/X* hypertrophic zone aRNA. Plots depict mean fold differences with standard deviation from the mean, N = 3, statistical significance was determined using Student’s *t* test, ** *p* < 0.01, *** *p* < 0.001, **** *p* < 0.0001.

Since cartilage-specific inactivation of XBP1 was shown to have a negligible effect on the severity of the disease phenotype in our MCDS mouse models, it follows that genes important to the pathology of MCDS must be regulated by the UPR independently of XBP1. Conversely, those genes whose expression is regulated in the chondrocyte UPR in an XBP1-dependent manner must not contribute to the overall disease pathology. To assign genes to XBP1-independent or XBP1-dependent cohorts, we conducted expression profiling of RNA derived from hypertrophic zones microdissected from our mutant and wildtype mice. Samples were initially validated by qPCR using *Agc1*, *Ctgf*, and *Matn1* as markers of cartilage extracellular matrix, and *Creld2*, *Derl3*, *Ero1l*, *Fgf21*, *Steap1*, and *p58* ^*IPK*^ as markers of the hypertrophic chondrocyte UPR [12]. *Agc1*, *Ctgf*, and *Matn1* were down-regulated (Fig 4A–4C) and *Creld2*, *Derl3*, *Ero1l*, *Fgf21* and *Steap1* were up-regulated (Fig 4D–4H) in *ColX* ^*N617K*^ and in *C/X* compared to their respective controls, suggesting that the downstream consequences of the collagen X-induced cartilage UPR are similar in the absence of an active XBP1 pathway. *Creld2* and *Derl3* were both significantly upregulated in *ColX* ^*N617K*^ compared with *C/X*, indicating that these genes are at least partially XBP1-dependent. A comparable pattern was observed for *Fgf21* and *Steap1*, however the difference between *ColX* ^*N617K*^ and *C/X* was not statistically significant. One UPR gene, *p58* ^*IPK*^, was up-regulated in *ColX* ^*N617K*^ but not in *C/X* (Fig 4I), consistent with its known role as a direct downstream target of XBP1 signalling [19]. This further confirms the lack of XBP1 signalling in the *C/X* cartilage.

**Fig 5:**
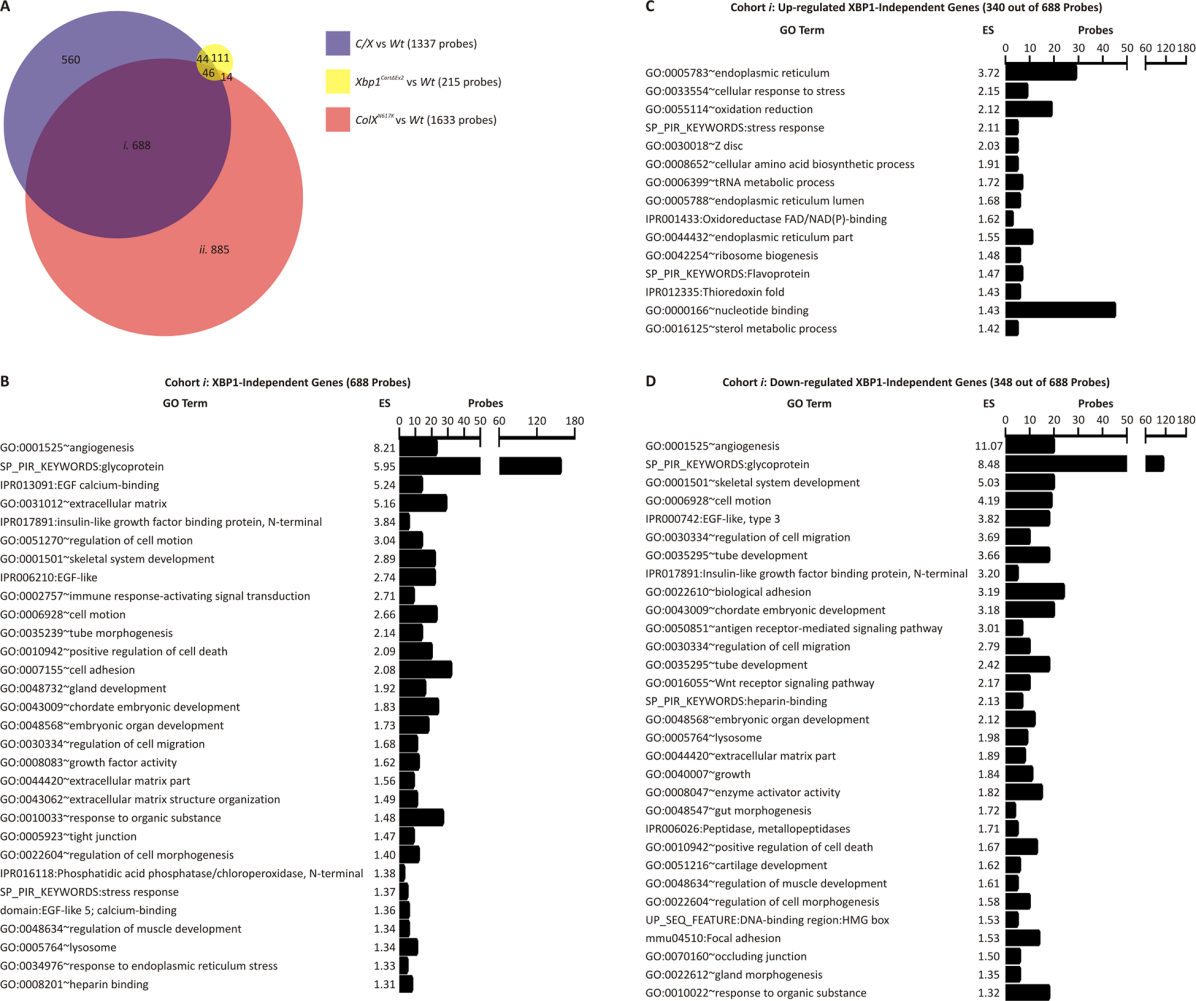
Microarray analysis of mutant and wildtype hypertrophic zones. *(A)* Venn diagram depicting the relationship between probes indicating differential gene expression (fold difference ≥ 2.0, adjusted *p* value ≤ 0.01) following comparisons of *C/X* versus wildtype (*Wt*) (blue), *Xbp1* ^*CartΔEx2*^ versus *Wt* (yellow), and *ColX* ^*N617K*^ versus *Wt* (red), by whole genome microarray analysis of hypertrophic zone aRNA. *(B-D)* Ontological analysis of *(B)* all probes in cohort *i* in *(A)*, or those showing *(C)* up-regulation or *(D)* down-regulation, by Functional Annotation Clustering, using DAVID v6.7 software, and depicting representative gene ontology terms from each annotation cluster achieving an enrichment score (ES) ≥ 1.3.

To explore XBP1-independent or XBP1-dependent dysregulated genes in more detail transcriptomic analysis was performed by interrogating these samples with whole genome microarrays. Initially, we performed separate comparisons of *C/X*, *Xbp1* ^*CartΔEx2*^, or *ColX* ^*N617K*^ with wildtype to identify microarray probes showing greater than two-fold differential expression and with an adjusted *p* value of ≤0.01 in each mutant compared to wildtype. By these criteria, differential expression was detected with 1337 probes for *C/X* versus wildtype (S1 Table), 215 probes for *Xbp1* ^*CartΔEx2*^ versus wildtype (S2 Table), and 1633 probes for *ColX* ^*N617K*^ versus wildtype (S3 Table). Subsequently each of these sets of differentially expressed genes were compared with one another as shown in the Venn diagram in Fig 5A. Of the 1337 probes with differential expression between *C/X* and wildtype, 688 were differentially expressed in *C/X* versus wildtype and *ColX* ^*N617K*^ versus wildtype but not in *Xbp1* ^*CartΔEx2*^ versus wildtype (Fig 5A, cohort *i*). These probes represent genes regulated in the chondrocyte UPR independently of XBP1. Of the 1633 probes indicating differential expression between *ColX* ^*N617K*^ versus wildtype, 885 were differentially expressed in *ColX* ^*N617K*^ versus wildtype but not in *Xbp1* ^*CartΔEx2*^ versus wildtype or *C/X* versus wildtype (Fig 5A, cohort *ii*), representing genes regulated in the chondrocyte UPR in an XBP1-dependent manner.

Ontological analysis revealed that the XBP1-independent cohort (Fig 5A, cohort *i*) was enriched with probes corresponding to genes associated with angiogenesis, glycoproteins, the extracellular matrix, the endoplasmic reticulum, and positive regulation of apoptosis (Fig 5B). Dysregulated apoptosis was confirmed by TUNEL analysis (Fig 3). We next partitioned the 687 probes of cohort *i* into those representing up-regulated genes or down-regulated genes in the collagen X mutants versus wildtype. The sub-cohort of 340 probes up-regulated independently of XBP1 was enriched with the majority of probes from cohort *i* corresponding to the endoplasmic reticulum, oxidation and reduction, and mRNA translation (tRNA metabolism and amino acid biosynthesis; Fig 5C). The sub-cohort of 348 probes down-regulated independently of XBP1 was enriched with the majority of probes from cohort *i* associated with angiogenesis, glycoproteins, the extracellular matrix, and skeletal system development (Fig 5D), consistent with the qPCR validation data for markers of ER stress, cartilage ECM (Fig 4) and angiogenesis, *Vegfa* and *Hbb-b1* (S2A and S2B Fig). Thus the XBP1-independent consequences of the chondrocyte UPR are two-fold, both designed to restore ER homeostasis. On the one hand, they include the transcriptional up-regulation of genes encoding components of the cell that support protein folding. On the other, they involve transcriptional down-regulation of genes encoding secreted proteins, many of which include glycoproteins, components of the cartilage extracellular matrix, and pathways involved in skeletal system development.

The XBP1-dependent cohort (Fig 5A, cohort *ii*) was enriched with probes corresponding to genes associated with organelle lumen, mitochondria, RNA processing and the cytoskeleton (S1A Fig). We partitioned the 885 probes of cohort *ii* into those representing genes up-regulated or down-regulated in response to ER stress in an XBP1-dependent manner. The sub-cohort of 479 probes up-regulated in *ColX* ^*N617K*^ versus wildtype in an XBP1-dependent manner was enriched with the majority of probes from cohort *ii* associated with organelle lumen (which includes ER-related genes), mitochondria, the nucleolus, and non-coding RNA processing and metabolism (S1B Fig). qPCR validation of the up-regulation of several ER genes (*Creld2*, *Derl3*, *Ero1l* and *p58IPK*) is shown in Fig 4. The sub-cohort of 406 probes down-regulated in *ColX* ^*N617K*^ versus wildtype in an XBP1-dependent manner was enriched with the majority of probes from cohort *ii* corresponding to the cytoskeleton, extracellular matrix, vasculature development, and ossification (S1C Fig). By qPCR we validated the down-regulation of several genes related to ossification, *Pthr1* and *Bmp8a* (S2C and S2D Fig). Thus as with the XBP1-independent consequences of the chondrocyte UPR, XBP1-dependent gene expression changes include the transcriptional up-regulation of genes encoding components of the cell that support ER homeostasis, and the transcriptional down-regulation of genes encoding membrane-bound and secreted proteins that are trafficked through the ER for post-translational modification and assembly.

### The developmental arrest of ER-stressed *ColX* ^*N617K*^ chondrocytes is regulated independently of XBP1

**Fig 6:**
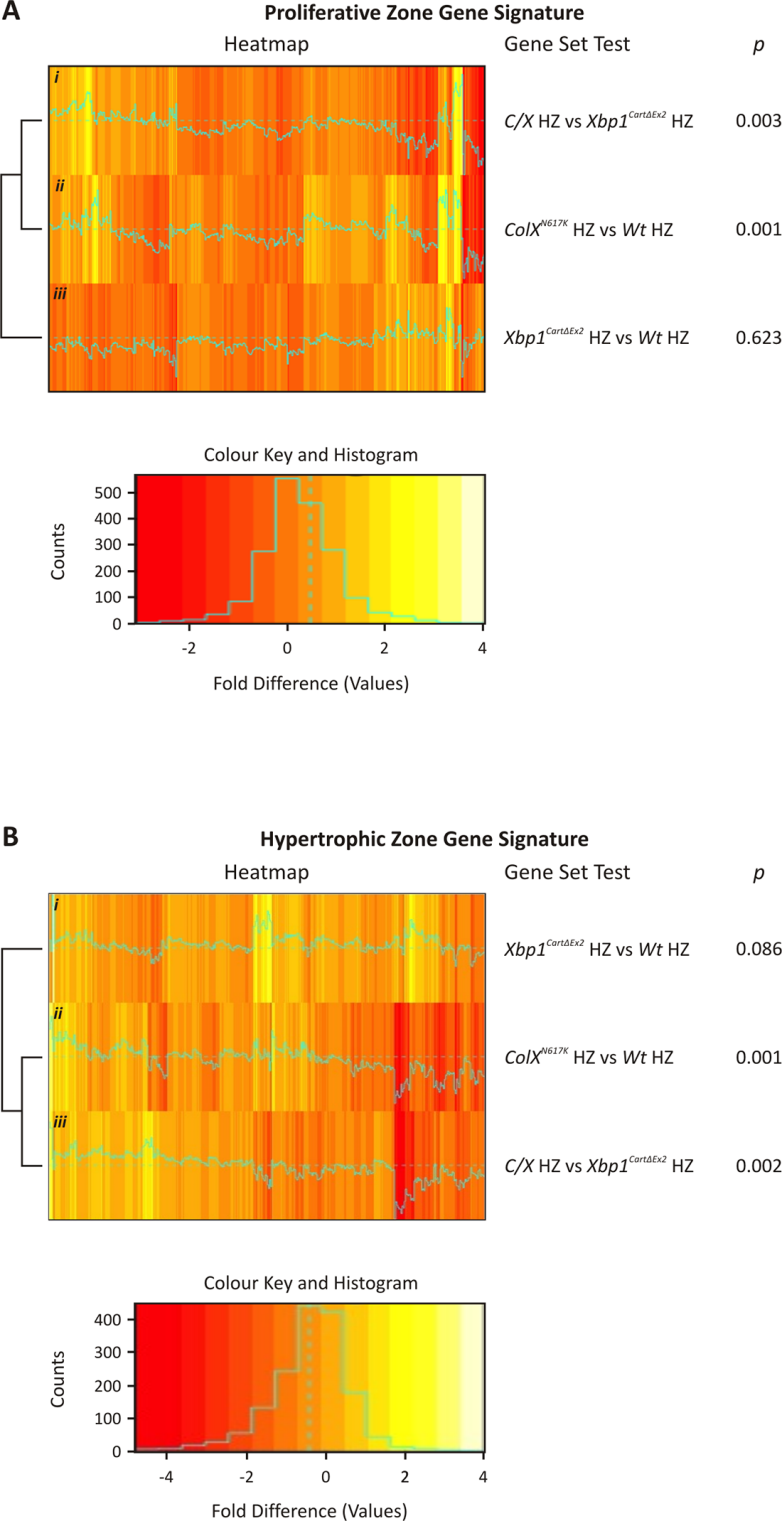
Expression of wildtype growth plate zone gene signatures in *ColX* ^*N617K*^, *Xbp1* ^*CartΔEx2*^, and *C/X* hypertrophic zones. Heatmaps depicting the relative fold difference (log fold change) of microarray probes representing *(A)* 773 wildtype (*Wt*) proliferative zone signature genes and *(B)* 510 *Wt* hypertrophic zone signature genes following the comparison of *C/X* versus *Xbp1* ^*CartΔEx2*^, *ColX* ^*N617K*^ versus *Wt*, and *Xbp1* ^*CartΔEx2*^ versus *Wt* hypertrophic zones; N = 3. For both heatmaps, each *Wt* growth plate zone signature gene is represented by a single bar, colour-coded according to relative expression as indicated, with up-regulated probes coloured yellow, and down-regulated probes coloured red.

We have previously shown that ER stress in the growth plate hypertrophic zone disrupts chondrocyte differentiation such that there is a delay in both the down-regulation of the proliferative chondrocyte gene expression signature, and up-regulation of the hypertrophic chondrocyte gene expression signature in mouse models of MCDS, including *ColX* ^*N617K*^ [12]. To determine whether XBP1 contributes to this disruption, we performed gene set tests comparing the differential expression of probes representing previously established [12] proliferative zone signature genes (Fig 6A) or hypertrophic zone signature genes (Fig 6B) in *ColX* ^*N617K*^ hypertrophic zones versus wildtype, *C/X* hypertrophic zones versus *Xbp1* ^*CartΔEx2*^, and in *Xbp1* ^*CartΔEx2*^ hypertrophic zones versus wildtype. Significantly elevated expression of the proliferative zone gene signature was observed in *C/X* versus *Xbp1* ^*CartΔEx2*^ (Fig 6Ai), as in *ColX* ^*N617K*^ versus wildtype (Fig 6Aii), but not in *Xbp1* ^*CartΔEx2*^ versus wildtype (Fig 6Aiii). Likewise we demonstrated significantly reduced expression of the hypertrophic zone gene signature in *C/X* versus *Xbp1* ^*CartΔEx2*^ (Fig 6Biii), similar to *ColX* ^*N617K*^ versus wildtype (Fig 6Bii), but not in *Xbp1* ^*CartΔEx2*^ versus wildtype (Fig 6Bi). These results indicate that the disrupted differentiation observed in chondrocytes expressing misfolding protein in the hypertrophic zone is caused by an XBP1-independent aspect of the chondrocyte UPR.

### Evidence for post-transcriptional inhibition of C/EBP-β-mediated gene expression in *ColX* ^*N617K*^ and *C/X* hypertrophic zones

Several recent studies have implicated C/EBP-β in regulating the transition of chondrocytes from proliferation to hypertrophy [16–18]. Moreover, GADD45-β [20,21] and RUNX2 [17] have been identified as transcriptional co-factors of C/EBP-β required for full induction of the hypertrophy program. It has also been established that the function of C/EBP transcription factors, including C/EBP-β, may be inhibited through interaction with the ER stress-responsive transcription factor, CHOP [22].

**Fig 7:**
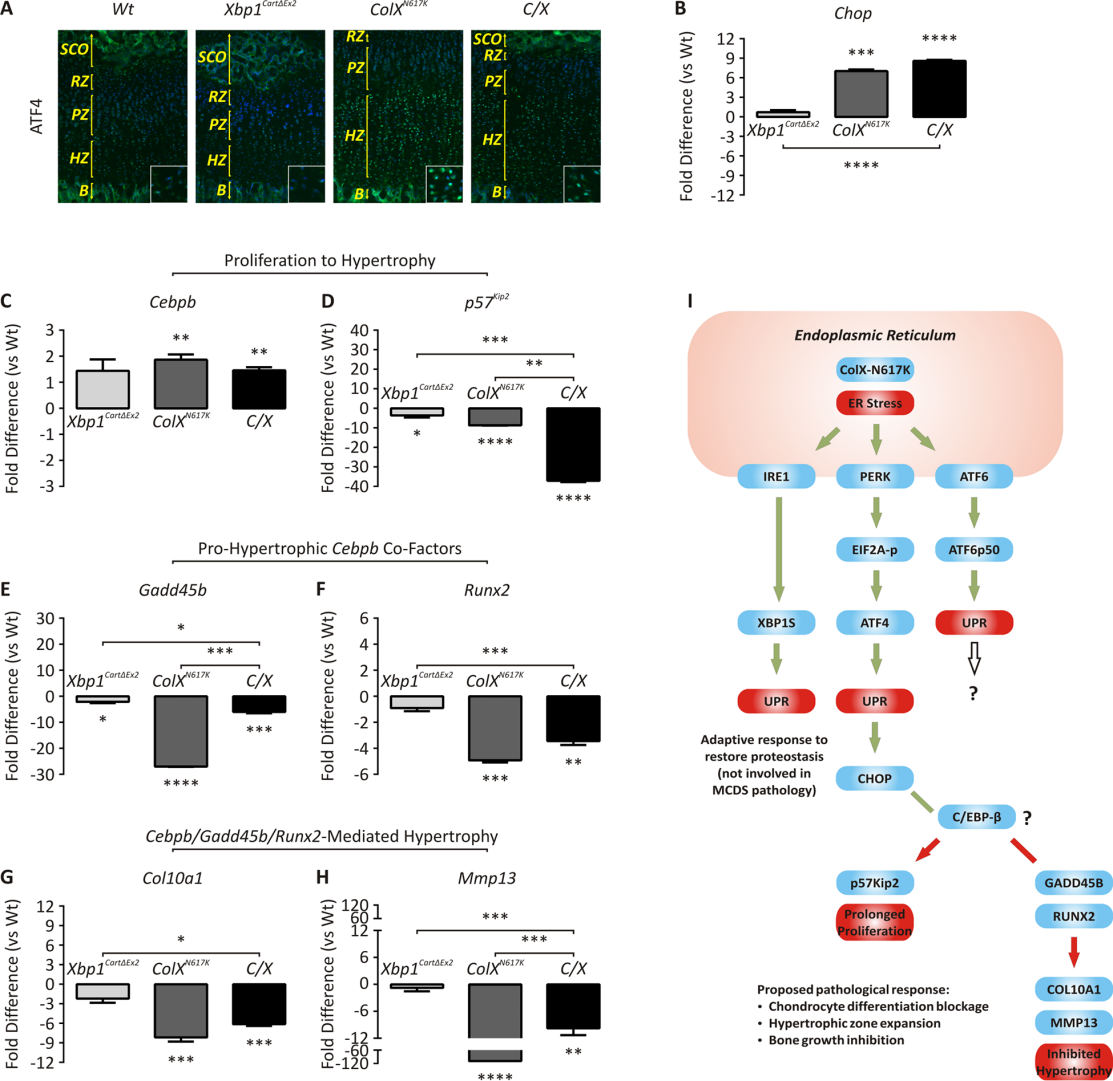
Dysregulated expression of genes involved in ER stress and chondrocyte differentiation. *(A)* Immunofluorescent analysis for ATF4 in tibial epiphyseal cryosections from 2 week wildtype (*Wt*), *Xbp1* ^*CartΔEx2*^, *ColX* ^*N617K*^ and *C/X* mice; B—Bone; HZ—Hypertrophic Zone; PZ—Proliferative Zone. *(B-H)* qPCR with primers specific for *(B) Chop*, *(C) Cebpb*, *(D) p57* ^*Kip2*^, *(E) Gadd45b*, *(F) Runx2*, *(G) Col10a1*, and *(H) Mmp13* on cDNA derived from *Wt*, *Xbp1* ^*CartΔEx2*^, *ColX* ^*N617K*^ and *C/X* hypertrophic zone aRNA. Plots depict mean fold differences with standard deviation from the mean; N = 3; statistical analysis performed using Student’s *t* test, * *p* < 0.05, ** *p* < 0.01, *** *p* < 0.001, **** *p* < 0.0001. *(I)* Schematic diagram of proposed model to explain the molecular pathology of MCDS. Blue boxes depict genes. Red boxes depict biological processes. Green arrows depict activation or up-regulation. Red arrows depict inactivation or down-regulation. Green lines depict increased interaction between proteins. Red lines depict decreased interaction between proteins.

Therefore we investigated whether the blockage in chondrocyte differentiation observed in our *ColX* ^*N617K*^ and *C/X* mice could be caused by inhibition of the transcriptional activity of C/EBP-β (Fig 7). By immunofluorescent analysis of wildtype and mutant growth plates, we confirmed that ATF4, a marker of PERK activation required for ER stress-responsive expression of CHOP [15], was up-regulated in the *ColX* ^*N617K*^ hypertrophic zone compared with wildtype, and in the *C/X* hypertrophic zone compared with *Xbp1* ^*CartΔEx2*^ (Fig 7A). Accordingly, by qPCR analysis of microdissected mutant and wildtype hypertrophic zones, we also confirmed up-regulation of *Chop* in *ColX* ^*N617K*^ versus wildtype, and in *C/X* versus *Xbp1* ^*CartΔEx2*^ (Fig 7B). Further qPCR analysis of the same samples was performed to establish expression profiles for *Cebpb*, *Gadd45b*, and *Runx2*, as well as C/EBP-β transcriptional targets, *p57* ^*Kip2*^, *Col10a1*, and *Mmp13* (Fig 7C–7H). *Cebpb* expression was up-regulated in *ColX* ^*N617K*^ versus wildtype, but differential expression was not observed between *C/X* and *Xbp1* ^*CartΔEx2*^. *p57* ^*Kip2*^, *Runx2*, *Col10a1*, and *Mmp13* were all down-regulated in both *ColX* ^*N617K*^ versus wildtype, and *C/X* versus *Xbp1* ^*CartΔEx2*^. *Gadd45b* was significantly downregulated in *ColX* ^*N617K*^ versus wildtype; in *C/X* versus *Xbp1* ^*CartΔEx2*^ downregulation of *Gadd45b* did not reach statistical significance. The qPCR expression profiles were broadly consistent with our microarray data for the same genes (S1 and S3 Tables). Overall our results support the hypothesis that C/EBP-β transcriptional activity is inhibited as a result of post-transcriptional inhibition of C/EBP-β, rather than reduced expression of *Cebpb* mRNA, coupled with ER stress-dependent down-regulation of C/EBP-β transcriptional co-factors, GADD45-β and RUNX2.

## Discussion

We and others have previously demonstrated that ER stress induced by expression of misfolding proteins in the mouse growth plate hypertrophic zone is sufficient to phenocopy MCDS [11]. Characterization of the molecular pathology of these mouse models of MCDS demonstrated that a canonical UPR is initiated involving activation of each of the canonical ER stress sensors that ultimately impairs bone growth by disrupting chondrocyte differentiation [11,12]. Here we demonstrate surprising redundancy of the IRE1/XBP1 signaling pathway in the MCDS UPR by showing that ablation of XBP1 signaling from chondrocytes in a mouse model of MCDS has no effect on the overall severity of the disease phenotype.

It has been reported previously that by comparison with ATF6 and PERK, the XBP1 pathway regulates the differential expression of only a small subset of ER stress-responsive genes in mammalian cells [23,24]. This raises the question of what purpose the IRE1/XBP1 pathway serves in the cartilage UPR, and what genes it controls. To assess the contribution of the IRE1/XBP1 pathway to the MCDS UPR, we used a transcriptional profiling approach to interrogate gene expression in hypertrophic zones microdissected from our MCDS mutant mouse models. We identified 886 probes indicating significant differential gene expression between *ColX* ^*N617K*^ and wildtype in an XBP1-dependent manner. Given the impact of XBP1 at the transcriptional level when activated in response to chondrocyte ER stress and the effects its activation can have on the secretory capacity of the cell, and considering that cartilage-specific inactivation of XBP1 leads to a mild dwarfism characterized by hypertrophic zone shortening [14], it is surprising that XBP1 is pathologically redundant in MCDS.

In addition to regulating the activity of XBP1, IRE1 activated during ER stress may also influence gene expression by degrading transcripts encoding membrane-bound and secreted proteins through regulated IRE1 dependent decay (RIDD) [25] and promote apoptosis via phosphorylation of JNK [26]. Of the established mammalian targets of RIDD, only *Scara3* and *Sparc* were downregulated in both *ColX* ^*N617K*^ versus wildtype (S1 Table) and *C/X* versus *Xbp1* ^*CartΔEx2*^ (S3 Table), suggesting that RIDD does not have a significant role in the pathology of MCDS. Whether or not JNK is phosphorylated during ER stress in *ColX* ^*N617K*^ or *C/X* chondrocytes is unknown. Nevertheless, our study is the first to reveal dysregulated cell death as a feature of the pathology of MCDS. Previously we [12] and others [27] reported that apoptosis was not increased above wildtype levels in the hypertrophic zones of mouse models of MCDS up to 10 days of age. It is uncertain why a significant increase in the rate of ER stress-induced apoptosis was apparent in *ColX* ^*N617K*^ and *C/X* by two weeks of age but not earlier. A steady trajectory in the post-natal growth rate of the *ColX* ^*N617K*^ mouse was observed until 3 weeks of age, where it increased markedly to reach its peak between three to four weeks [11]. Thus, it is unlikely that the delay in ER stress-induced apoptosis in these mice until two weeks of age is attributable to significant growth-related increases in ER protein load at this time. An alternative possibility is that the delay might coincide with increased physical activity of the mice, implying that the fate of ER-stressed chondrocytes in the *ColX* ^*N617K*^ and *C/X* hypertrophic zones may be influenced by biomechanical force. Favouring a mechanism involving the combined influence of ER stress and biomechanical strain, we observed skewing in the antero-posterior distribution of apoptotic chondrocytes in the hypertrophic zones of both *ColX* ^*N617K*^ and *C/X* such that significantly more apoptotic chondrocytes were present closer to the periosteum than in the central portion of the growth plate. Moreover the bowing of weight-bearing long bones and *coxa vara* observed in human MCDS and mouse models [28] also points towards biomechanical stresses influencing endochondral bone development in this disease, in addition to ER stress. The onset of hypertrophic zone expansion and ER stress well before the increase in apoptosis in MCDS [11,12,27,29] indicates that ER stress-induced cell death is not a central feature of the disease pathology. Moreover, our data suggest that the proposal that the UPR in MCDS is adaptive and permits cell survival by alleviating the stress [27] may be restricted to early stages of cartilage development and maturation.

We and others have demonstrated previously that ATF6 is proteolytically cleaved [11] and eIF2α phosphorylated [12] in mouse models of MCDS, implying activation of ATF6 and PERK respectively. Our transcriptomic analyses (S1 and S3 Tables) corroborate these findings, with ATF6 targets including *BiP*, *Creld2*, *Derl3*, and *Grp94* [30], PERK/ATF4/CHOP targets including *Aldh1l2*, *Aldh18a1*, *Angptl6*, *Clcn3*, *Cdsn*, *Cyb5r1*, *Cxad2*, *Erlin1*, *Fads3*, *Gpt2*, *Hspa9*, *Iars*, *Leprotl1*, *Mthfd2*, *Nars*, *Otub2*, *Ppp1r15a*, *Sars*, *Slc7a11*, *Steap1*, *Vldlr*, *Wars*, *Xpot*, and *Zfp238* [8,31], and *Erp72*, *Grp58*, *Herpud1*, and *Pig-A*, which are recognised as targets of both ATF6 and PERK, all upregulated in *ColX* ^*N617K*^ and *C/X* versus wildtype. Thus, we cannot definitively discriminate the relative contributions of ATF6 versus PERK to the IRE1/XBP1-independent effects of ER stress in MCDS chondrocytes, since both pathways seem to be activated strongly.

We identified a cohort of 688 probes indicating XBP1-independent differential gene expression between our collagen X mutant mice and their respective controls. As with the XBP1-dependent cohort, several subsets were associated with processes and machinery designed to improve protein folding. Conspicuously, we also identified large subsets associated with angiogenesis, glycoproteins and the extracellular matrix. Subsequent partitioning of the XBP1-independent probes into subsets corresponding to up-regulated genes or down-regulated genes revealed that most of the genes associated with organelles or functions predicted to enhance protein folding were up-regulated in *ColX* ^*N617K*^ and *C/X* compared with controls, while most of the genes encoding markers of angiogenesis, glycoproteins, extracellular matrix molecules, and proteins associated with skeletal system development were down-regulated in the collagen X mutants compared with their respective controls. As with previous studies proposing UPR-mediated transcriptional suppression of physiological gene networks as a critical consequence of ER stress in other disease contexts [32–34], so too our results suggest a pattern of ER stress-induced disruption to growth plate maturation in MCDS through transcriptional suppression of genes encoding secreted proteins, including components of the cartilage extracellular matrix.

Several lines of evidence point towards the UPR interfering with chondrocyte differentiation by post-translational interruption of C/EBP-β. Various studies have implicated C/EBP-β as a key regulator in the transition of chondrocytes from proliferation to hypertrophy. *Cebpb* ^-/-^ mice were characterized by dwarfism involving elongation of the growth plate proliferative zone and delayed chondrocyte hypertrophy [16]. Proliferative zone elongation in these mice was due to reduced expression in the pre-hypertrophic zone of *p57* ^*Kip2*^, a gene identified as a transcriptional target of C/EBP-β that encodes a cyclin-dependent kinase inhibitor important for the exit of chondrocytes from cell division [16,35]. In addition to driving the expression of *p57* ^*Kip2*^, C/EBP-β represses the expression of *Sox9* and *Col2a1*, both important markers of chondrocyte proliferation [18]. Thus, C/EBP-β appears to have dual roles as a transcription factor controlling chondrocyte proliferation, switching off the expression of genes involved in maintaining the proliferative phenotype and switching on the expression of genes involved in terminating chondrocyte proliferation.

As well as promoting the exit of chondrocytes from their proliferative program, C/EBP-β also actively promotes the entry of chondrocytes into hypertrophy. It has been shown that C/EBP-β co-localizes in the growth plate hypertrophic zone with GADD45-β and collagen X [20], and that it acts cooperatively with GADD45-β to regulate *Col10a1* and *Mmp13* expression [20,21]. MMP13 is critical for endochondral ossification, since *Mmp13*-null mice are characterized by hypertrophic zone expansion, reduced collagen turnover, and delayed ossification [36]. In addition to GADD45-β, RUNX2 has also been implicated as a transcriptional co-factor of C/EBP-β. The *Cebpb* ^-/-^ mouse dwarfism phenotype was significantly exacerbated when crossed with a heterozygous *Runx2* knockout mouse to generate *Cebpb* ^-/-^;*Runx2* ^+/-^, in which impaired cartilage remodelling through loss of *Mmp13* expression resulted in elongation of the hypertrophic zone, in addition to the elongated proliferative zone seen in *Cebpb* ^-/-^ [17]. Thus, C/EBP-β actively promotes chondrocyte hypertrophy and growth plate matrix remodelling and turnover by interacting cooperatively with GADD45-β and RUNX2 to drive the expression of key markers of terminal chondrocyte maturation including *Col10a1* and *Mmp13*.

Histomorphometric and expression profiling data in this and previous studies [11,12,27] are consistent with inhibition of C/EBP-β activity in *ColX* ^*N617K*^ and *C/X* growth plates. The hypertrophic zone expansion we have observed in *ColX* ^*N617K*^ [11,12] and *C/X*, the manner in which growth plate zone gene signatures were dysregulated in *ColX* ^*N617K*^ and *C/X*, and the down-regulation of key C/EBP-β transcriptional targets, *p57* ^*Kip2*^, *Col10a1*, and *Mmp13* observed here and previously [27] are all highly reminiscent of the skeletal phenotypes reported for the *Cebpb* ^-/-^ and *Cebpb* ^-/-^;*Runx2* ^+/-^ mice [16,17]. Moreover, the mis-expression of SOX9 and *Col2a1* in the 13del collagen X transgenic mouse is consistent with suppressed C/EBP-β activity in the MCDS growth plate [27]. Crucially however, the expression of *Cebpb* itself was not significantly down-regulated in the hypertrophic zones of either *ColX* ^*N617K*^ or *C/X*, implying that disruption to C/EBP-β activity in these mice must have occurred post-transcriptionally. The down-regulation of *Gadd45b* and *Runx2* that we observed in *ColX* ^*N617K*^ and *C/X* relative to their controls is expected to have depleted the availability of C/EBP-β transcriptional co-factors required to promote hypertrophy in these mutants, and may thus have contributed to the elongation of their hypertrophic zones, as well as the dwarfism characteristic of each model. Nevertheless, that neither *Runx2* ^+/-^ nor *Cebpb* ^*+/-*^;*Runx2* ^*+/-*^ mice display a skeletal phenotype [17] suggests that partial depletion of hypertrophy-dependent C/EBP-β transcriptional co-factors alone is insufficient to disrupt bone growth in the manner we have observed in *ColX* ^*N617K*^ and *C/X*. Rather, it suggests that loss of C/EBP-β activity itself, is also required. Moreover, while the depletion of GADD45-β and RUNX2 coupled with inactivation of C/EBP-β may have contributed to the delayed onset of chondrocyte hypertrophy, there is no evidence to suggest that it is in any way connected with the reduction in *p57* ^*Kip2*^ expression, and associated mis-expression of proliferative zone signature genes that we observed in the *ColX* ^*N617K*^ and *C/X* hypertrophic zones. Thus it would appear that besides the depletion of GADD45-β and RUNX2, the UPR must interfere with C/EBP-β activity by other means as well.

Another mechanism by which C/EBP-β activity might be impaired in the growth plates of *ColX* ^*N617K*^ and *C/X* mice is through interaction with CHOP, which was induced in both mutants as a consequence of increased ATF4 expression. It has been shown that the transcriptional activity of C/EBP transcription factors can be attenuated by CHOP [22]. Other work has demonstrated that a consequence of prolonged UPR activation resulting from chemically induced ER stress in mice compromised by genetic inactivation of either of various components of the UPR, is transcriptional suppression of metabolic gene expression networks in the liver leading to hepatic steatosis, at least partially through the inhibition of C/EBP-α by CHOP [33,34]. These studies provide a precedent for disrupted cell differentiation *in vivo* by inhibition of C/EBP proteins by CHOP following ER stress. Thus we expect that in addition to ER stress-induced down-regulation of *Gadd45b* and *Runx2*, inhibition of C/EBP-β by CHOP probably contributes to the down-regulation of C/EBP-β transcriptional targets we observed in *ColX* ^*N617K*^ and *C/X*. Expression of CHOP is understood to be a later event in the kinetics of UPR activation [37]. Supporting this, *in situ* analysis of UPR protein expression in the 13del collagen X transgenic mouse indicated that CHOP is activated relatively late in the MCDS UPR, after the onset of stress indicated by expression of the spliced form of XBP1 at the top of the mutant hypertrophic zone [27]. The delayed inhibition of C/EBP-β following the onset of ER stress may explain the observation from several MCDS mouse models that hypertrophic differentiation begins to proceed briefly before being interrupted and appearing to revert in response to UPR induction [11,27].

In all, these findings have led us to propose a model to explain the pathology of MCDS as follows. The onset of chondrocyte hypertrophy in MCDS is marked by the expression of misfolding collagen X, leading to ER stress. Each of the canonical ER stress-sensing pathways is activated. Through the combined, XBP1-independent effects of ER stress-induced up-regulation of CHOP and down-regulation of *Gadd45b* and *Runx2*, C/EBP-β transcriptional activity is inhibited. Consequently, the stressed chondrocytes maintain or reactivate the expression of proliferative chondrocyte markers, and fail to express several key markers of terminal chondrocyte maturation, leaving the cells in a proliferative chondrocyte-like state. The collective consequences of these inputs are disruption to cartilage remodelling, vascularisation, and mineralisation, leading to hypertrophic zone expansion and dwarfism. This is the first study to our knowledge to provide evidence consistent with disruption to C/EBP-β-mediated gene transcription by ER stress in the pathology of a model of human disease. Moreover, it adds to the growing body of evidence arguing for the importance of CHOP in modulating the expression of physiological gene networks regulated by C/EBP transcription factors during ER stress [32–34].

## Materials and Methods

### Generation of *C/X* mice

*Col10a1* p.Asn617Lys mice (*ColX* ^*N617K*^) [11] were crossed with mice in which *Xbp1* mRNA is inactivated by the Cre recombinase-mediated deletion of exon 2 in *Col2a1*-expressing cells (*Xbp1* ^*CartΔEx2*^) [14] to generate the compound mutant, *Col10a1* p.Asn617Lys/*Xbp1* ^*CartΔEx2*^ (*C/X*). These mice were viable, fertile and bred normally and were housed under pathogen-free conditions. All animal studies were approved by the Murdoch Childrens Research Institute Animal Ethics Committee (Approval numbers AEC#718 and #787). Genotyping was performed as previously described [11,14]. As previously [14], RT-PCR and sequencing were subsequently performed on cartilage RNA as described to confirm deletion of *Xbp1* exon 2 in *C/X* chondrocytes.

### Skeletal preparations and morphometry

Morphometric analyses were performed on skeletal preparations following Alcian blue/ Alizarin red staining as described [14].

### Histology and immunofluorescence

Histology was performed on 10μm neutral buffered formalin-fixed cryosections of proximal tibial epiphyses from two week old wildtype, *ColX* ^*N617K*^, *Xbp1* ^*CartΔEx2*^, or *C/X* mice. Toluidine blue staining [12] and immunofluorescent analyses using antibodies specific for collagen II or collagen X [14] were performed as described. Immunofluorescent analysis of ATF4 expression was performed using 1:100 rabbit anti-human ATF4 antibody (D4B8; Cell Signaling Technology) and an appropriate fluorescent secondary antibody (10μg/ml; Molecular Probes, Life Technologies), as follows. Prior to antigen retrieval, all sections were incubated for 10 min at room temperature in PBS, 0.2% Triton X-100 (Sigma-Aldrich). For ATF4 antigen retrieval, sections were incubated for 10 min at room temperature in 1% SDS (Sigma Aldrich) in PBS. All immunofluorescence sections were counterstained and mounted using VECTASHIELD Mounting Medium with DAPI (Vector Laboratories, Inc), and visualized by fluorescent microscopy with an Axio Imager M1 fluorescent microscope (Zeiss).

### Cell death

TUNEL was performed with the In Situ Cell Death Detection Kit, Fluorescein (Roche) to detect DNA fragmentation in cells undergoing programmed cell death as described [12].

### Microdissection of hypertrophic zones, RNA isolation and amplification

Hypertrophic zones were microdissected, and RNA isolated and amplified from one proximal tibial growth plate from each of three two week old wildtype, *ColX* ^*N617K*^, *Xbp1* ^*CartΔEx2*^, and *C/X* as described [14]. The yield and integrity of all samples were validated as appropriate with a Qubit 2.0 fluorometer (Invitrogen), Nanodrop 1000 spectrophotometer, or a 2200 TapeStation (Agilent Technologies), using a High Sensitivity R6K Screen Tape Kit (Agilent Technologies). Each RNA validation procedure was performed according to the relevant manufacturer’s specifications.

### Expression profiling by qPCR and mouse whole genome microarray analysis

Quantitative PCR (qPCR) was performed on equal quantities of cDNA derived from tibial growth plate hypertrophic zone aRNA from two week old wildtype, *ColX* ^*N617K*^, *Xbp1* ^*CartΔEx2*^, and *C/X* mice using the LightCycler 480 Probes Master Kit (Roche Applied Science) on a LightCycler 480 II qPCR machine (Roche Applied Science) as described [12]. All aRNA samples were interrogated by microarray analysis using single-colour hybridisations to MouseWG-6_V2 whole genome microarrays according to the manufacturer’s specifications (Illumina).

Microarrays were analysed using the package Limma [38] from the statistical language R. Background correction and quantile normalization were performed using the necq function. Probes that did not have above background expression in at least three samples were removed from the analysis after normalisation. Probes that were classified as “bad” or “no match” from *illuminaMousev2*.*db* [39] were also removed. Self-contained gene set testing was performed using the ROAST [40] from the Limma package. Heatmaps were generated using heatmap.2 from the gplots R package.

### Statistical methods

All quantitative data were generated as stated in figure legends. All analyses were conducted using GraphPad QuickCalcs software (La Jolla, CA, USA).

## Supporting Information

S1 Fig Ontological analysis of XBP1-dependent genes. *(A)* all probes in cohort *ii* in Fig 5A, or those showing *(B)* up-regulation or *(C)* down-regulation, by Functional Annotation Clustering, using DAVID v6.7 software, and depicting representative gene ontology terms from each annotation cluster achieving an enrichment score (ES) ≥ 1.3.
(TIF)

S2 Fig Quantitative PCR of mutant and wildtype hypertrophic zones. qPCR with primers specific for *(A) Vegfa*, *(B) Hbb-b1*, *(C) Pthr1*, and *(D) Bmp8a* on cDNA derived from *Wt*, *Xbp1* ^*CartΔEx2*^, *ColX* ^*N617K*^, and *C/X* hypertrophic zone aRNA. Plots depict mean fold differences with standard deviation from the mean, N = 3, statistical significance was determined using Student’s *t* test, * *p* < 0.05, ** *p* < 0.01, *** *p* < 0.001, **** *p* < 0.0001.
(TIF)

S1 Table *C/X* vs *Wt* differentially expressed genes. (XLSX)

S2 Table *Xbp1* ^*CartΔEx2*^ vs *Wt* differentially expressed genes. (XLSX)

S3 Table *ColX* ^*N617K*^ vs *Wt* differentially expressed genes. (XLSX)
