# Supplementary figures and images for: XBP1-Independent UPR Pathways Suppress C/EBP-β Mediated Chondrocyte Differentiation in ER-Stress Related Skeletal Disease
Source: PLoS Genet. 2015 Sep 15;11(9):e1005505. doi: 10.1371/journal.pgen.1005505 (PMC4651170; doi:10.1371/journal.pgen.1005505)

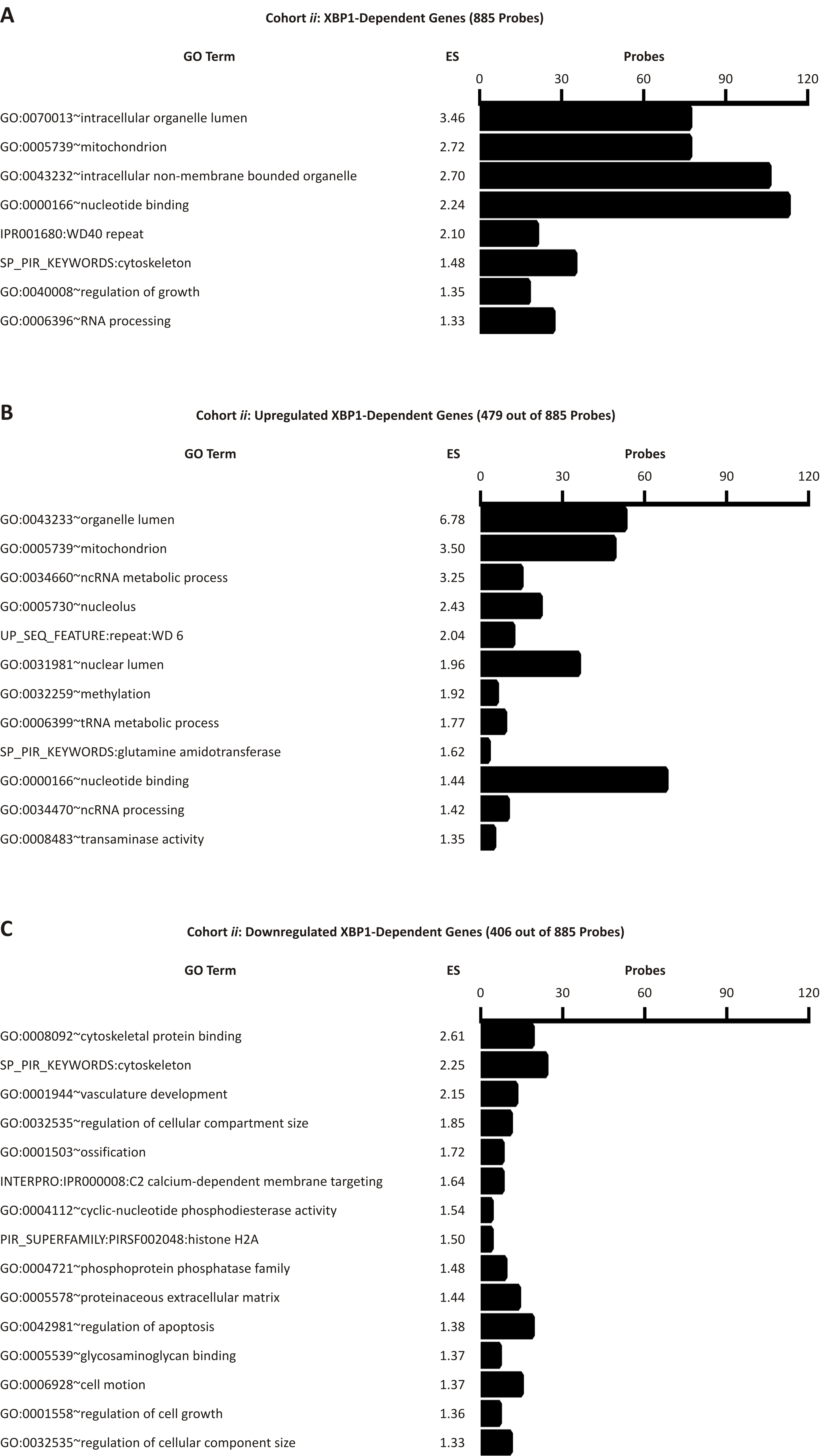

Supplement: S1 Fig — (A) all probes in cohort ii in Fig 5A, or those showing (B) up-regulation or (C) down-regulation, by Functional Annotation Clustering, using DAVID v6.7 software, and depicting representative gene ontology terms from each annotation cluster achieving an enrichment score (ES) ≥ 1.3. (TIF) [file pgen.1005505.s001.tif]

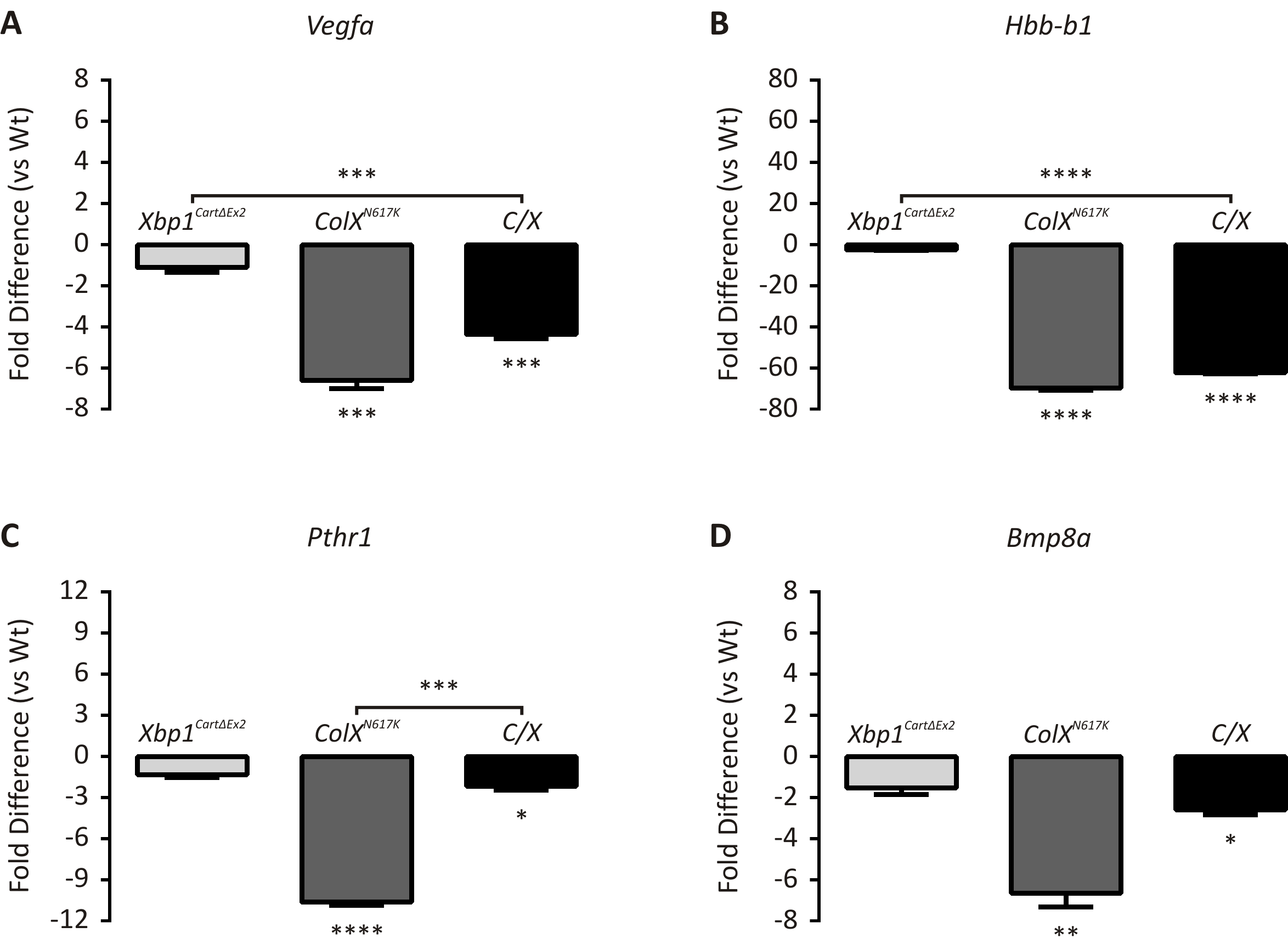

Supplement: S2 Fig — qPCR with primers specific for (A) Vegfa, (B) Hbb-b1, (C) Pthr1, and (D) Bmp8a on cDNA derived from Wt, Xbp1 CartΔEx2, ColX N617K, and C/X hypertrophic zone aRNA. Plots depict mean fold differences with standard deviation from the mean, N = 3, statistical significance was determined using Student’s t test, * p < 0.05, ** p < 0.01, *** p < 0.001, **** p < 0.0001. (TIF) [file pgen.1005505.s002.tif]
